# Supplementary material for: Ginsenoside Re as a Probe for Evaluating the Catalytic Potential of Microcrystalline Cellulose for the Degradation of Active Pharmaceutical Ingredients
Source: Pharmaceuticals (Basel). 2025 Jun 11;18(6):869. doi: 10.3390/ph18060869 (PMC12195823; doi:10.3390/ph18060869)
Supplement: Supplementary file 1 [file pharmaceuticals-18-00869-s001.zip › pharmaceuticals-3675586-supplementary.pdf]

**Supplementary Data**

# Ginsenoside Re as a Probe for Evaluating the Catalytic Potential of Microcrystalline Cellulose for the Degradation of Active Pharmaceutical Ingredients

Xinyu Gao, Shengyuan Xiao *

Engineering Center of Edible and Medicinal Fungi, Ministry of Education, Jilin Agricultural University, Changchun,130118, China

E-mail addresses:

Gao X. 13188538499@163.com

* Corresponding author:

Xiao S. Prof., xiao03435@163.com, Tel: +86 431 84532953, Fax: +86 431 84532953.

### 1.1. Identification of the hydrolysis products of ginsenoside Re

Ginsenoside Re solution (or/and Ro), acid (or alkali) and pure water were put into a 10 mL glass vial and mixed well with vortexing. The vial was sealed and incubated in a 90 °C water bath for 6h, and then the vial was taken out and cooled under tap water. To guarantee all the products are dissolved well, the same volume of methanol was finally added into the vial. The suspension was then centrifuged and used for analysis. Hydrolyses of ginsenoside catalyse by MCCs were carried follow the procedure in the manuscript.

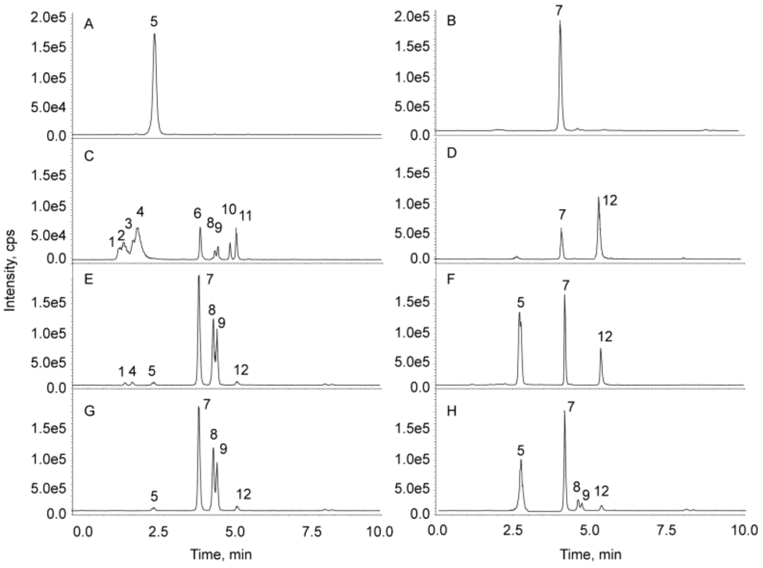


Figure S1. Representative chromatograms of ginsenoside Re, Ro, and their transformed products using alkali, acid, and MCCs. A, Chromatogram of Re; B, Chromatogram of Ro; C, Re hydrolysate catalyzed by 1% formic acid; D, Ginsenoside Ro transformed products in 1.0% sodium carbonate (w/v); E, Ginsenoside Ro and Re mixture transformed products in 0.5 ppm sulfuric acid (v/v); F, Ginsenoside Ro and Re mixture transformed products in 0.5 ppm sodium carbonate (w/v); G, and H, Ginsenoside Ro and Re mixture transformed products catalyzed by market available microcrystalline celluloses. The MS/MS data of these compounds were listed in Table S1. The identification of the products in the reaction was described as follows.

The identifications of the transformed products of Re and Ro were carried out under a negative Q3 scan mode. The scan range of the mass analyser is 400 – 1200 amu. The MS/MS data was obtained in a Q3 IDA (information-dependent acquisition) mode. The separation program and the condition of the mass spectrometer were described in the following section. Ginsenoside Re was observed to be unstable in acidic circumstances, but Ro is not stable in diluted alkali solution. Figure S1 shows the representative chromatograms of ginsenoside Re, Ro, and their transformed product under the treatment of diluted acid, sodium carbonate, and microcrystalline cellulose. The MS/MS data and identification of the products are summarized in Table S1.

The molecular weights of the aglycones of compounds 1 and 4 are both 494, which is 18 amu more than that of the panaxatriol. As transformed products of ginsenoside Re, their MS/MS data are consistent with the structure of ginsenoside Rf2 [15], a Δ (23, 24) hydration product of ginsenoside

Rg2 (Reaction 2). Compounds 1 and 4 were tentatively identified as 20(S) and 20(R)-ginsenoside Rf2 based on these observations.

Compounds 2 and 3 displayed similar dissociation patterns as compounds 1 and 4, but their molecular weights are 656, which is 146 less than that of compounds 1 and 4. Compounds 2 and 3 were tentatively identified as 20(S) and 20(R)-24-OH-ginsenoside Rh1, the Δ (23, 24) hydration products of ginsenoside Rh1 (Reaction 2).

The molecular weights of compounds 6, 8, 9, 10, and 11 are all 784. Compounds 8 and 9 were identified as 20(S) and 20(R)-ginsenoside Rg2 by their chromatographic retention time using reference material of Rg2. Others might be the dehydration products of ginsenoside Rf2 produced in a similar mechanism (Reaction 3) to the dehydration production of 20(Z/E)-ginsenoside F4 [16] and ginsenoside Rg6 [17]. Compounds 6, 10, and 11 were tentatively identified as 24-OH-ginsenoside Rg6, 20(Z)-24-OH-ginsenoside F4, and 20(E)-24-OH-ginsenoside F4.

The hydrolysis of ginsenoside Re in 1% formic acid solution can be summarized as follows: The hydrolysis of the glycosidic bond at C-20 produced 20(R/S)-ginsenoside Rg2 (Reaction 1, 8, and 9). The hydration of the Δ (23, 24) double bond of Rg2 gave 20(R/S)-ginsenoside Rf2 (Reaction 2, 1 and 4). The hydrolysis of the disaccharide bond of compounds 1 and 4 produced 2 and 3. The dehydration of the hydroxyl group at C-20 of compounds 1 and 4 produced compounds 6, 10, and 11 (Reaction 3). Strangely, the dehydration products of Rg2 (20(R/S)-F4 and Rg6) have not been observed under the conditions of this experiment.

Ginsenoside Ro is unstable in an alkaline circumstance (Figure S1, D). Nearly 60% of Ro hydrolyzed to produce zingibroside R1 after 6h of heating (90 °C) in 1.0% sodium carbonate solution.

The glycosidic bond at C-20 of Re can be hydrolyzed by very diluted sulfuric acid (Figure S1, E); however, Ro displayed acidic resistance (Figure S1, E). What is different from the reaction of Re in 1.0% formic acid, the transformed products of Re in diluted sulfuric acid were mainly Rg2 (Figure S1, E). On the other hand, the ester bond at C-28 of Ro can be hydrolyzed by diluted alkaline conditions, and ginsenoside Re was observed to be quite stable under weak alkaline circumstances (Figure S1, F).

Ginsenoside Re was observed to be hydrolyzed by commercially available MCCs (Figure S1, G and H) to produce 20(R/S)-ginsenoside Rg2. The dehydration of C20-OH and the hydrolysis of other glycosidic bonds have not been observed.

Table S1. Identification of transformed products of ginsenoside Re and Ro

| No | Rt/min | MS | MS/MS | Identification |
|---|---|---|---|---|
| 1 | | 847 [M+HCOO$^-$], 801 [M-H]$^-$ | 801 [M-H]$^-$; 655[M-H-146]$^-$; 637[M-H-164]$^-$; 493[M-H-146-162]$^-$ | 20(S)-ginsenoside Rf2 [1] |
| 2 | | 701 [M+HCOO$^-$], 655 [M-H]$^-$ | 655[M-H]$^-$; 493[M-H-162]$^-$ | 20(S)-24-OH-ginsenoside Rh1 |
| 3 | | 701 [M+HCOO$^-$], 655 [M-H]$^-$ | 655[M-H]$^-$; 493[M-H-162]$^-$ | 20(R)-24-OH-ginsenoside Rh1 |
| 4 | | 847 [M+HCOO$^-$], 801 [M-H]$^-$ | 801 [M-H]$^-$; 655[M-H-146]$^-$; 637[M-H-164]$^-$; 493[M-H-146-162]$^-$ | 20(R)-ginsenoside Rf2 [1] |

| | | | |
|---|---|---|---|
| 5 | 845 $[M+HCOO^{-}]$, 799 $[M-H]^{-}$ | 783 $[M-162]^{-}$; 637$[M-H-162-146]^{-}$ | Ginsenoside Re |
| 6 | 829 $[M+HCOO^{-}]$, 783 $[M-H]^{-}$ | 783 $[M-H]^{-}$; 637$[M-H-146]^{-}$; 619$[M-H-164]^{-}$; 475$[M-H-146-162]^{-}$; 391$[M-H-146-162-84]^{-}$ | 24-OH-ginsenoside Rg6 |
| 7 | 955 $[M-H]^{-}$ | 793 $[M-H-162]^{-}$; 631$[M-H-162-162]^{-}$ | Ginsenoside Ro |
| 8 | 829 $[M+HCOO^{-}]$, 783 $[M-H]^{-}$ | 783 $[M-H]^{-}$; 637$[M-H-146]^{-}$; 619$[M-H-164]^{-}$; 475$[M-H-146-162]^{-}$; 391$[M-H-146-162-84]^{-}$ | 20(S)-ginsenoside Rg2 |
| 9 | 829 $[M+HCOO^{-}]$, 783 $[M-H]^{-}$ | 783 $[M-H]^{-}$; 637$[M-H-146]^{-}$; 619$[M-H-164]^{-}$; 475$[M-H-146-162]^{-}$; 391$[M-H-146-162-84]^{-}$ | 20(R)-ginsenoside Rg2 |
| 10 | 829 $[M+HCOO^{-}]$, 783 $[M-H]^{-}$ | 783 $[M-H]^{-}$; 637$[M-H-146]^{-}$; 619$[M-H-164]^{-}$; 475$[M-H-146-162]^{-}$; 391$[M-H-146-162-84]^{-}$ | , 20(Z)-24-OH-ginsenoside F4 |
| 11 | 829 $[M+HCOO^{-}]$, 783 $[M-H]^{-}$ | 783 $[M-H]^{-}$; 637$[M-H-146]^{-}$; 619$[M-H-164]^{-}$; 475$[M-H-146-162]^{-}$; 391$[M-H-146-162-84]^{-}$ | 20(E)-24-OH-ginsenoside F4 |
| 12 | 793 $[M-H]^{-}$ | 631$[M-H-162]^{-}$ | Zingibroside R1 |

Reaction 1: Hydrolysis of the C20-glycosidic bond

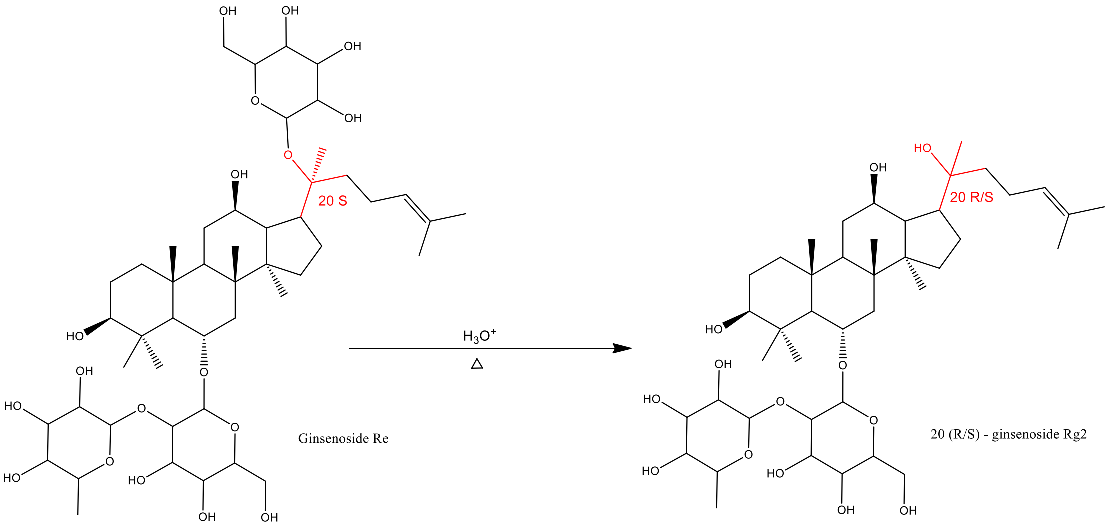


Reaction 2: Hydration of the Δ (23, 24) double bond

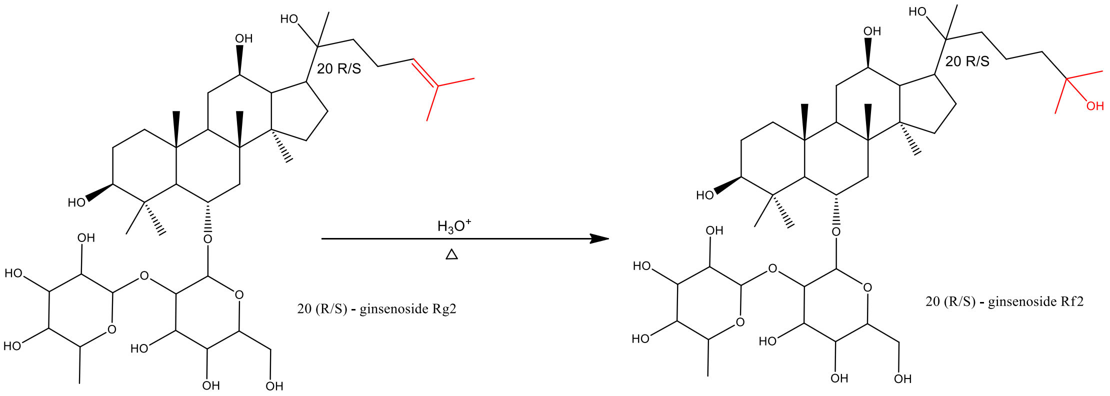


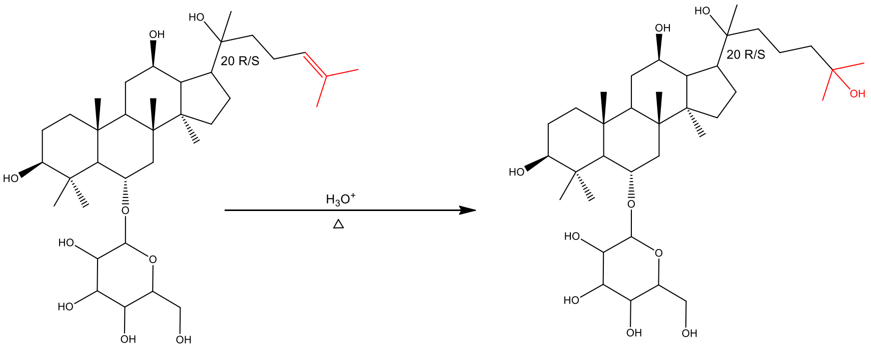


20 (R/S) - ginsenoside Rg2

20(R)-24-OH-
ginsenoside Rh1

## Reaction 3: Dehydration of C20-OH

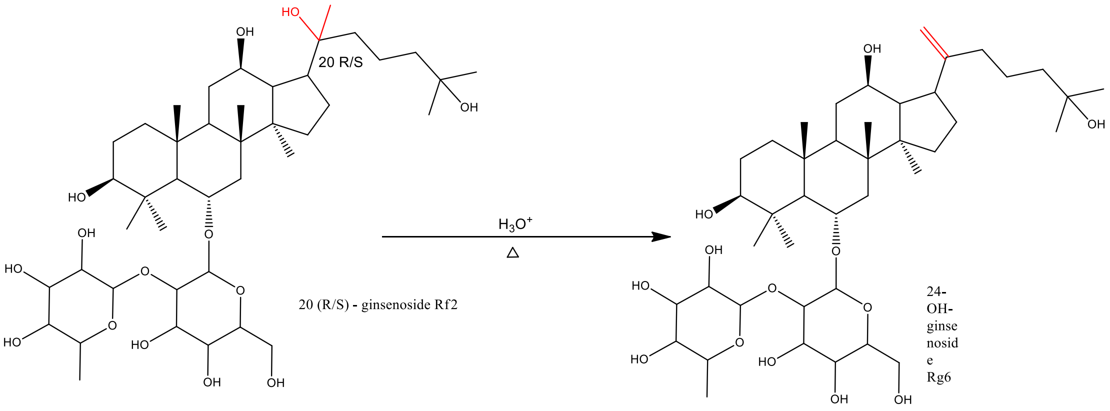


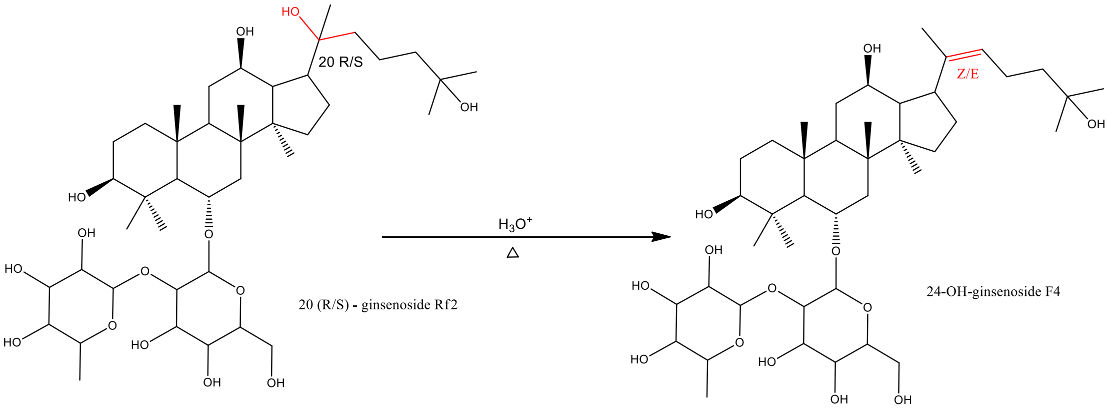

Reaction 4: Hydrolysis of ginsenoside Ro

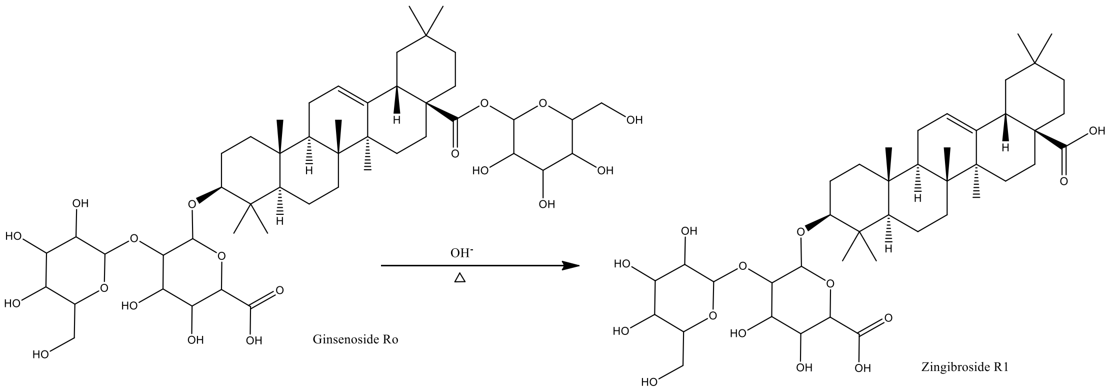


Figure S2 Chemical reactions of Ginsenoside Re in acidic solution and Ro in alkaline solution.

## 1.2. Determination of ginsenosides Re, Rg2, Ro and zingiberoside R1

The analyses were performed using an AB SCIEX 4500 LC MS/MS system consisting of a TripleQuad MS spectrometer and a SCIEX ExionLC system that includes a degasser, an auto-sampler, 2 pumps, a column oven, and a controller. The separation was performed using a Chromolith eRP C18 silicon column (50 × 2 mm, Merck, Germany). The mobile phase consisted of 3.0 mM ammonium hydroxide aqueous solution (A) and acetonitrile (B) in a gradient eluting programme. The gradient programme was as follows: 10% B was maintained for the first 2 minutes, followed by a linear gradient of 10 – 40% B for 2 – 5min, 40 – 50% B for 5 - 8 min, 50 – 70% B for 8 – 9 min, and 70 – 95%B for 9 – 12 min. The flow rate was 0.3 mL/min. The determination of ginsenosides was performed in negative MRM mode. The ion transmissions of different compounds were shown in Table S2. The ion source parameters were as follows: curtain gas, 35 psi; ion spray voltage, -4500 V; gas 1, 50 psi; gas 2, 60 psi; temperature of gas 2, 650 °C. The interface parameters were as follows: declustering potential, -120 V; enhancement potential, -10 V; collision cell exit potential, -15 V.

Table S2 MRM detection of ginsenoside Re, Ro and their transformed products

| No | Q1* | Q2 | Dwell, ms | Name | DP, v | CE, v |
|---|---|---|---|---|---|---|
| 1 | 459.4 | 375.4 | 2 | diol | -120 | -70 |
| 2 | 475.5 | 391.5 | 2 | triol | -120 | -70 |
| 3 | 945.3 | 945.3 | 2 | Re 0 | -120 | -20 |
| 4 | 945.4 | 799.4 | 2 | Re 1 | -120 | -48 |
| 5 | 945.4 | 637.4 | 2 | Re 2 | -120 | -55 |
| 6 | 945.4 | 619.4 | 2 | Re 4 | -120 | -55 |
| 7 | 945.4 | 475.4 | 2 | Re 5 | -120 | -66 |
| 8 | 799.41 | 637.4 | 2 | Rf 1 | -120 | -45 |
| 9 | 799.41 | 619.4 | 2 | Rf 2 | -120 | -45 |
| 10 | 799.41 | 475.4 | 2 | Rf 3 | -120 | -63 |
| 11 | 817.6 | 817.6 | 2 | Rf T 1 | -120 | -20 |
| 12 | 817.6 | 493.4 | 2 | Rf T 2 | -120 | -60 |
| 13 | 801.4 | 493.4 | 2 | Rf2 1 | -120 | -60 |
| 14 | 847.4 | 493.4 | 2 | Rf2 2 | -120 | -60 |
| 15 | 701.4 | 701.4 | 2 | Rf3 1 | -120 | -20 |
| 16 | 701.4 | 655.5 | 2 | Rf3 2 | -120 | -20 |
| 17 | 701.6 | 493.4 | 2 | Rf3 3 | -120 | -60 |
| 18 | 655.5 | 493.4 | 2 | Rf3 4 | -120 | -48 |
| 19 | 859.4 | 799.4 | 2 | Rg1 1 | -120 | -32 |
| 20 | 859.4 | 637.4 | 2 | Rg1 2 | -120 | -44 |
| 21 | 859.4 | 619.4 | 2 | Rg1 3 | -120 | -44 |
| 22 | 859.4 | 475.4 | 2 | Rg1 4 | -120 | -56 |
| 23 | 799.4 | 637.4 | 2 | Rg1 5 | -120 | -36 |
| 24 | 799.4 | 619.4 | 2 | Rg1 6 | -120 | -36 |
| 25 | 799.4 | 475.4 | 2 | Rg1 7 | -120 | -50 |
| 26 | 843.6 | 697.4 | 2 | Rg1 Ac | -120 | -20 |
| 27 | 847.4 | 639.2 | 2 | Rg1 H2 1 | -120 | -64 |
| 28 | 847.4 | 477.4 | 2 | Rg1 H2 2 | -120 | -66 |
| 29 | 847.6 | 801.4 | 2 | Rg1 H2 3 | -120 | -20 |
| 30 | 783.4 | 783.4 | 2 | Rg2 0 | -120 | -20 |
| 31 | 783.4 | 637.4 | 2 | Rg2 1 | -120 | -45 |
| 32 | 783.4 | 475.5 | 2 | Rg2 3 | -120 | -45 |
| 33 | 825.6 | 783.6 | 2 | Rg3 Ac 1 | -120 | -40 |
| 34 | 825.6 | 765.4 | 2 | Rg3 Ac 2 | -120 | -40 |
| 35 | 829.6 | 783.6 | 2 | Rg3 Ad | -120 | -20 |
| 36 | 765.6 | 619.6 | 2 | Rg6 | -120 | -60 |
| 37 | 765.6 | 603.6 | 2 | Rg5 | -120 | -60 |
| 38 | 765.6 | 765.6 | 2 | Rg6 0 | -120 | -20 |
| 39 | 807.4 | 765.6 | 2 | Rg6 Ac | -120 | -40 |
| 40 | 637.4 | 475.4 | 2 | Rh1 1 | -120 | -37 |
| 41 | 637.4 | 391.4 | 2 | Rh1 2 | -120 | -60 |

| | | | | | | |
|---|---|---|---|---|---|---|
| 42 | 683.6 | 637.4 | 2 | Rh1 ad | -120 | -20 |
| 43 | 621.3 | 459.3 | 2 | CK,h2 | -120 | -40 |
| 44 | 667.4 | 459.4 | 2 | CK,h2 1 | -120 | -60 |
| 45 | 667.4 | 621.4 | 2 | CK,h2 2 | -120 | -20 |
| 46 | 955.5 | 955.5 | 2 | Ro 0 | -120 | -20 |
| 47 | 955.4 | 793.4 | 2 | Ro 1 | -120 | -65 |
| 48 | 955.4 | 749.4 | 2 | Ro 2 | -120 | -66 |
| 49 | 955.4 | 731.3 | 2 | Ro 3 | -120 | -73 |
| 50 | 955.4 | 613.3 | 2 | Ro 4 | -120 | -75 |
| 51 | 955.4 | 455.2 | 2 | Ro 5 | -120 | -84 |
| 52 | 793.5 | 793.5 | 2 | zR1 0 | -120 | -20 |
| 53 | 793.5 | 631.5 | 2 | zR1 1 | -120 | -48 |
| 54 | 617.5 | 455.4 | 2 | OA-glu | -120 | -40 |

*, all possible products have been taken in account

The linearity was generated using the MS/MS signal of the ion channels as fellow: 945.5/945.5 (DP, 120 v and CE 20 v) for ginsenoside Re; 783.4/783.4 (DP, 120 v and CE 20 v) for ginsenoside Rg2; 955.5/955.5 (DP, 120 v and CE 20 v) for ginsenoside Ro and 793.4/793.4 (DP, 120 v and CE 20 v) for zingibroside R1. These signals showed good linearity against the amount. The linear relationship of the detector response against the amount of Re was y=105,774.6x-1,835.7, $R^2$=0.99981 within the range 0.05-3.00 ng/inj, while that for Rg2 was y=1,868,168.5x+36,264.5, $R^2$=0.99825 for 0.04-2.48 ng/inj. The linearity of the detector response against the amount for Ro was y=407,894.4x+9,174.9, $R^2$=0.99969 within the range of 0.01-3.00 ng/inj, and that for zR1 was y=5,765,527.6x-170,951.0, $R^2$=1.00000 within the range of 0.01-2.49 ng/inj.

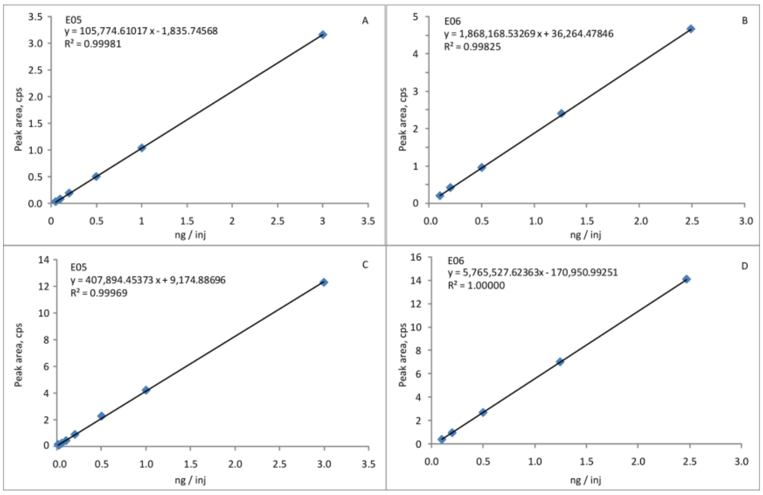


Figure S3 Plot of the linearity of ginsenoside Re, Rg2, Ro and zingibroside R1

The recoveries of the analytes were calculated using spiked MCC. Briefly, 200 mg MCC was added to a 10 mL vial, and then 100 µl ginsenoside solution (1 mg/mL) and 1 mL of pure water were added and mixed well with vortexing. Then, 1 mL of methanol was added to the vial and mixed vigorously by vortexing. Approximately 1 mL of suspension was taken out and centrifuged at 10,000 rpm for 5 min. the supernatant was taken out for analysis. The same volume of analyte solution was mixed with 2 mL solvent (Methanol: water = 1:1) to be used as a reference. The recovery was calculated by comparing the peak area of an analyte in the spiked MCC to that in the reference sample. The recovery was 100.18±1.56% for ginsenoside Re, and 98.32±1.86 for ginsenoside Rg2, 98.09±3.34% for ginsenoside, Ro and 92.71±2.83 for zingibroside R1.

## References

15. Park JD, Lee YH, Kim SI. Ginsenoside Rf2, a new dammarane glycoside from Korean red ginseng (Panax ginseng). Archives of Pharmacal Research. 1998 October 01; 21(5):615-7.
16. Ryu J-H, Park J-H, Kim T-H, Sohn DH, Kim JM, Park JH. A genuine dammarane glycoside, (20E)-ginsenoside F4 from Korean red ginseng. Archives of Pharmacal Research. 1996 August 01; 19(4):335.
17. Yang XW LL, Tian JM, Zhang ZW, Ye JM, Gu WF. Ginsenoside-Rg 6, a novel triterpenoid saponin from the stem-leaves of Panax ginseng C. A. Mey. Chinese Chemical Letters. 2000; 11:909-12.
